# Quantifying the transmissibility of human influenza and its seasonal variation in temperate regions

**DOI:** 10.1371/currents.RRN1125

**Published:** 2010-06-13

**Authors:** James Truscott, Christophe Fraser, Wes Hinsley, Simon Cauchemez, Christl Donnelly, Azra Ghani, Neil Ferguson, Aronrag Meeyai

**Affiliations:** ^*^MRC Centre for Outbreak Analysis and Modelling, Dept. of Infectious Disease Epidemiology, Imperial College London; ^†^Imperial College London; ^‡^MRC Centre for Outbreak Analysis and Modelling, Department of Infectious Disease Epidemiology, Imperial College London; ^¶^MRC Centre for Outbreak Analysis and Modelling, Imperial College London; ^#^MRC Centre for Outbreak Analysis & Modelling, Imperial College London; ^**^Director, MRC Centre for Outbreak Analysis and Modelling, Imperial College London and ^††^MRC Centre for Outbreak Analysis & Modelling, Imperial College

## Abstract

Seasonal influenza has considerable impact around the world, both economically and in mortality among risk groups. The long term patterns of disease are hard to capture with simple models, while the interplay of epidemiological processes with antigenic evolution makes detailed modelling difficult and computationally intensive. We identify a number of characteristic features of flu incidence time series in temperate regions, including ranges of annual attack rates and outbreak durations. We construct pseudo-likelihoods to capture these characteristic features and examine the ability of a collection of simple models to reproduce them under seasonal variation in transmission. Results indicate that an age-structured model with non-random mixing and co-circulating strains are both required to match time series data. The extent of matching behaviour also serves to define informative ranges for parameters governing essential dynamics. Our work gives estimates of the seasonal peak basic reproduction, R0, in the range 1.7-2.1, with the degree of seasonal variation having limited impact of these estimates. We find that it is only really possible to estimate a lower bound on the degree of seasonal variation in influenza transmissibility, namely that transmissibility in the low transmission season may be only 5-10% less than the peak value. These results give some insight into the extent to which transmissibility of the H1N1pdm pandemic virus may increase in Northern Hemisphere temperate countries in winter 2009. We find that the timescale for waning of immunity to current circulating seasonal influenza strain is between 4 and 8 years, consistent with studies of the antigenic variation of influenza, and that inter-subtype cross-immunity is restricted to low levels.

## Introduction

Seasonal influenza causes significant levels of morbidity and mortality around the world each year, yet its dynamics and the annual sequence of pathogen subtypes are hard to predict [1]. Understanding the mechanisms underlying the annual behaviour of influenza and their sensitivity to parameters is important for the forecasting and control of seasonal epidemics and also in assessing the effect of the introduction of novel antigenic strains. The recent epidemic of a novel H1N1 strain is a topical example [2]. The initial epidemic occurred in spring and summer, ‘out of season’ in the Northern hemisphere. Uncertainty with regard to intensity of transmission during summer greatly adds to the difficulty in forecasting the behaviour of the virus in the winter. 

Many respiratory transmissible diseases exhibit seasonal behaviour. This seasonal forcing is well known for causing a wide range of oscillatory behaviour, as illustrated by incidence rates for measles and pertussis [3] [4] [5]. The source of seasonal forcing is largely attributed to annual variations in the intensity of contact between people, but a range of other causes have been proposed. In the case of influenza, variation in vitamin D levels and air humidity have recently been suggested [6] [7]. In the case of influenza, dynamics are additionally complicated by both antigenic drift and shifts and the presence of multiple antigenically distinct subtypes. These mechanisms combine to generate complex seasonal behaviour, featuring a range of annual attack rates and epidemic durations and alternating sequences of dominant annual strains [8]. Detailed and computationally-intensive simulations are required to fully integrate the epidemiological and genetic aspects of long term behaviour [9] [10]. 

In this paper, we identify a set of key features characterizing seasonal influenza time series, expressing them as a series of pseudo-likelihoods. Using these likelihoods, we investigate what mechanisms are essential in a simple transmission model to reproduce observed behaviour and quantify the parameter ranges for which this is so. In particular we concentrate on the form of the seasonally varying transmission rate and the mean duration of immunity to the currently circulating influenza strain. We do not address the mechanisms behind  seasonality or antigenic evolution, but examine how they contribute to epidemiological dynamics and parameterize and quantify their influence. 

## Methods

 The time series for influenza incidence rate are highly irregular over time and, between different populations, reporting rates and methods of data collection vary. However, a number of key features and patterns can be identified and used to characterize seasonal flu incidence in temperate countries of the Northern hemisphere: 

      Epidemic duration. The vast majority of seasonal flu outbreaks are observed between the extremes of mid-November and the end of April [8]. Since a background rate of incidence is present throughout the year, duration is difficult to define precisely. The range of values quoted for the duration of an annual epidemic (ADE) is typically between 3 and 16 weeks [1] [11]. We discuss  ranges of values and definitions in more detail in Supplementary Information . 

      Attack rate. The proportion of the population infected in a year, which we term the annual attack rate (AAR), is very difficult to determine, as a significant proportion of infections will be asymptomatic and only a proportion of symptomatic cases will seek healthcare. Hence sentinel data on influenza like illness (ILI) can only give relative information, such as the fractional variation in incidence over time. Data from France and the UK (See Figure 1 )  indicate a standard deviation for AAR across successive years of approx 40% of the mean attack rate [12] [13]. Results from serological and virus isolation data from closely monitored populations indicate a mean AAR  of approximately 10% for seasonal influenza, rising to 30% or more for influenza pandemics [10] [12].  Information on consultation rates for reinforce known cases also suggests a figure of 10% (See Supplementary Information ). 

      Periodic behaviour. As its name implies, seasonal flu is characterized by annual outbreaks in temperate countries, as distinct from measles, which exhibited biennial behaviour prior to the introduction of immunisation. However, the pattern of influenza incidence doesn’t repeat strictly each year; there is considerable variation in the size and timings of annual epidemics. Hence within the range of types of periodic behaviour shown by the simple deterministic models we examine, those of most interest are not purely annual, but retain some variation from year to year and so fall into the category of ‘chaotic’. 

      Strain variation. Virus isolation studies show that individual seasonal epidemics are usually dominated by a single type and/or subtype, although others may be present at low levels [14]. Successive seasons are usually dominated by different types and subtypes, although often the same strain is present for several seasons (See Figure 1 ). 

To capture these features in the output of our models, we define pseudo-likelihoods on the series of epidemic durations and AARs for successive years. We assume normal distributions for both durations and epidemics and AARs and then construct pseudo-likelihood that the output from the model is drawn from the target distributions (See Supplementary Information ).

- We explore parameter space to identify regions of highest likelihood. Although a simplified description of the epidemiological and evolutionary mechanisms of the flu, our model nevertheless contains a large number of parameters. We concentrate on the following groupings.
- The seasonal peak value of *R*_0_, here termed *R*_p_, and its relative amplitude* δ* (*R*_p_(1-*d*) being the seasonal minimum value of *R*_0_). These parameters control the seasonal forcing of the model;

      The timescale for the generation of antigenically new strains, represented by  mean duration of immunity to the current flu strain, *D* [see Model Section].

- External force of infection, ε, representing effect of contact between members of the population and infected individuals in other countries.
- The degree of assortativity in the contact patterns between children and adults, θ.

The values and sources of other parameters are listed in Table 1 .  

## Model

 We use a deterministic SIRS compartmental model , to which we have added a number of refinements to capture critical aspects of influenza infection and immunity. The model structure is laid out in detail in Supplementary Information; here we discuss only the most important features.   The effect of seasonal forcing is included through a time-varying contact parameter 

\begin{equation*}\beta (t)={{\beta }_{m}}(1+\Delta \beta \cos (2\pi t))\end{equation*}

However, we express this variation in terms of peak *R*_0_ and relative amplitude of variation throughout. In exploring values for these parameters, we compare with the range of estimates that exist for *R*_0_. The majority of these are calculated for the major global epidemics (1918, 1957, 1968), since the antigenic novelty of pandemic viruses means an assumption of a serologically naïve population can be made, making analysis simpler. For seasonal flu, knowledge of population susceptibility is necessary to estimate *R*_0_ (as opposed to the effective reproduction number, *R*). Estimated values for the 1918 pandemic range from 1.3 to 2.8 [15]. Similar values are found for the ’57 and ’68 epidemics [16]. These values also correspond well with those from studies of seasonal flu, giving winter *R*_0_ of 1.7 and school holiday *R*_0_ of 1.4 [11].

We represent the generation time for the pathogen by an Erlang distribution with shape parameter *k*=4 and mean 1/α, where α=2.2 days [17] [18]. Our model incorporates 2 strains of influenza, for instance representing H1N1 and H2N3, to try and capture aspects of the co-circulation of multiple influenza types and subtypes [8]. To represent the immune status of individuals with regard to the strains, we use the multistrain formulation introduced by Gogg and Swinton, in which individuals are classified according to their current immune status [19]. There are, therefore, 4 states for individuals in the model; entirely susceptible, immune to either strain 1 or 2 and immune to both strains. The formulation allows for the inclusion of a basic cross-immunity mechanism, whereby an individual infected with either strain has a probability, φ, of becoming immune to the other as well (assuming that this was not already the case) The flow between different immune states is illustrated in Figure 2 . We note that this cross-immunity response is different from the short-term non-specific response with regard to influenza strains considered elsewhere [9], though cross-immunity is assumed to wane at the same rate as strain-specific immunity.

Our model also includes a mechanism for loss of immunity, returning individuals to a susceptible state. Antigenically, flu strains can be grouped into clusters within each of which there is a high level of cross-immunity, but between which cross immunity is much lower or absent [20]. The appearance of a new cluster therefore corresponds to a step change in the susceptibility of the population to the current strain. Our model caricatures this process with a time-scale, *D*, for resistant individuals to become susceptible to the current strain again. As antigenically distinct clusters appear every 2-8 years [21] [22], this is our expected range for values of *D*.

There is evidence from contact studies and from flu incidence modelling, that infectious contact between individuals is highly assortative and age dependent, with the highest rates among school-age children [11] [23] [24]. We include these effects by stratifying the population into children (<14 years) and adults (> 14 years) and employing a mixing matrix to describe contact between the two groups. The degree of assortativity is controlled by the parameter, θ, and can be varied between random mixing (θ=0), where groups contact each other proportional to the fraction of the population they represent, and wholly assortative (θ=1) where each group mixes only with itself. Differences in intensity of contact are captured by relative susceptibility and infectiousness parameters, ρ and ψ (See Supplementary Information for details).

Simulations were run using a population of 60 million, approximating the population of the UK. Due to the large population, a deterministic deterministic model was used. Tests using the corresponding stochastic implementations showed no significant variation from the deterministic results and the presence of a continuous low level external force of infection precluded the possibility of extinction.

## Results

 Figure 3 shows the variation in the pseudo likelihoods as a function of peak value and amplitude of *R*_0_ (Other parameters as given in Table 1 ). It is clear that the attack rate experienced by the population and, to a lesser extent, the periodicity of the seasonal epidemic are strongly dependent on the peak value of *R*_0_, but largely independent of the amplitude of the transmission coefficient. Likelihood values greater than -100 correspond to regions in which realistic behaviour is found. A region of high likelihood exists for values of peak *R* between 1.6 and 2 and relative amplitude > 5% , with the highest likelihoods found around δ=0.2. The likelihood drops rapidly for smaller amplitudes as variation in contact rate is no longer sufficient to stimulate short duration epidemics. 

The position of the high likelihood band in Figure 3 is dependent on both the timescale for the loss of immunity, *D*, and the nature of the mixing between children and adults, as controlled by the parameter *θ*. The rate at which immunity is lost is the main balance against loss of susceptibles through infection. As a result, both the AAR and the periodicity of the time series are directly affected by this parameter (see Supplementary Information ). Dependence on *D* is illustrated in Figure 4 , which shows an optimal region for model behaviour in the region *1.5<R*_p_<2.0 and *D*>3.5. 

 Figure 5 shows a set of time series generated for a range of amplitudes with *R*_p_=2 (other parameters as in Table 1 ). This range lies largely within the ‘acceptable’ likelihood window in Figure 3 . For δ>0.05 (Fig. A), outbreak durations fall outside the acceptable range, giving *LL* < -100. Time series B is representative of the highest likelihood regions (*LL* > 40). The model captures the annual nature of  outbreaks as well as the variation in attack rate that occurs between years. For δ=0.2, the AAR varies in the range 6-14% with a regular alternating sequence of subtypes. Larger amplitudes (Fig. C and D) generate episodes of more extreme behaviour, featuring large epidemics (AAR ≈25%) followed by years with negligible activity and the breakdown of regular antiphase behaviour in the two strains. These patterns  are characterized by intermediate likelihoods (-100 < *LL* < -40).

The mixing parameter, θ, allows the host population contact patterns to vary between wholly assortative (θ=1) and random mixing (θ=0). Increasing the assortativity of mixing concentrates infections more strongly in age groups, decreasing the available susceptibles and hence the attack rate. The effect is comparable to the restriction of susceptibles as the immunity timescale, *D*, is increased. The periodic behaviour of the model as a function of *R*(*t*) is largely independent of mixing (See Figure 6 ). As a result, a range of values of *D* and *θ* have very similar likelihoods and generate similar behaviour. Figure 7 shows the periodicity and likelihood surfaces as  functions of *D* and *θ* for peak *R*_0_ =2 with a broad diagonal region of high likelihood in which increased mixing compensates for slower return of immune individuals to the susceptible class. 

The cross-immunity parameter, *φ*, allows us to vary the interaction of the two strains for complete independence (φ=0) to one in which infection with either renders the victim immune to both. The latter extreme is very similar to having only a single strain (See Supplementary Information). Figure 8 shows a parameter scan across values of *φ* and *D*, for *R*_p_=2. Clearly there is a range of possible combinations of *φ* and *D* generating high likelihoods. This correlation arises for reasons analogous to those governing the correlation of *θ* and *D*. Cross-immunity increases the effective loss of susceptibles caused by any single infection event and hence reduces the attack rate. This effect can be offset by a reduced immune period recycling susceptibles more quickly. Unlike for *θ*, changes in *φ *induce a change in the cyclical dynamics of the system, confining the high likelihood region to *φ* <0.4. 

 The behaviour of our model is quite sensitive to the assumed external force of infection, ε. The level of external forcing discussed in the Methods section is negligible in comparison to the average force of infection generated by the indigenous population. Since low prevalences of infected individuals are associated with long-period and chaotic solutions for SIR models, this level of importation of infectives strongly encourages annual and biennial behaviours and removes highly chaotic solutions from the region of high likelihood. The effect of importation rate can be seen in Figure 9 . For external forces of infection above about 2.5e-5/year, only annual and biennial solutions are found to give high pseudo-likelihoods. Optimal behaviour is found for an external force of infection of approximately 3.2e-6/year. 

## Discussion

Our main observation is that the criteria for a realistic incidence time series as laid out in the Methods Section can be satisfied for realistic values of the *R*_0_ and the time-scale for turn-over of antigenically novel strains. As shown in Figure 3, acceptable behaviour is found for peak *R*_0_ values ranging from around 1.6 to 2.1. The amplitude of variation of *R*_0_ is much less constrained, however. Values of δ less than 0.1 fail to generate sufficiently temporally-localised epidemics. Appropriate time series (Fig.5 B-D) are found for values of δ greater than 0.1. These resolve into two qualitatively different forms: a high likelihood regular time series for values centred around δ =0.2 (Fig. 5B) and a lower likelihood episodic series interrupted by more unpredictable behaviour in terms of both attack rates and the sequence of strains (Fig. 5C and D). Both these types of behaviour can be seem in time-series data. A strong dependence on peak *R*_0_ is to be expected as it is during the period of peak *R* that the majority of infections that contribute to the annual attack rate occur. The weak dependence of model behaviour on the amplitude of *R*_0_ indicates that the seasonal incidence time series tells us little about the variation of infectious contact rate. This is particularly unfortunate in the context of forecasting the behaviour of the current novel H1N1 strain this winter. 

It is well know that inter-epidemic ‘trough’ prevalence levels have a strong influence on the periodic behaviour of temporally forced models and it might therefore be expected that the amplitude of variation would affect the behaviour of the model. However the presence of an external force of infection prevents prevalence falling to levels where this effect would be felt. It is certainly the case that for high values of importation rate (>3.2e-5/year), externally generated cases interfere sufficiently with the natural dynamics of the system to prevent the irregular behaviour seen in real data(see Figure 9). 

Looking at time series generated by the model with parameter values from within the maximum likelihood band, we can see two types of behaviour: strictly annually alternating dominant strains with a range of attack rates between 6-14% (δ=0.2) and periods of much higher variation in AAR during which the strict sequence of dominant strains break down. These two types of behaviour bracket the observed behaviour seen in French ILI data, for example [8]. 

The mechanisms of cross-immunity, loss of immunity and mixing dynamics are closely related through their effect on the balance of generation and consumption of susceptibles. Both decreased assortativity and increased cross-immunity cause a faster drain on the susceptible population and shorter immune periods increase their replenishment. As a result, it is not possible to give independent optimal values for these parameters. However, both cross-immunity and immune period cause changes in the periodic behaviour of the system and this allows some bounds to be set on values. Figure 4 shows that values of D < 3 years have very low likelihoods as the irregular behaviour that gives the most appropriate distribution of annual attack rate values is replaced by regular behaviour. This effect can be seen more clearly in Figure 7, which also indicates for D>7 years attack rates and behaviour rule out acceptable solutions. Similarly, for cross-immunity values greater than 0.4 generate inappropriate modes of annual behaviour or attack rates that are too small. 

Behaviour with respect to the parameters θ and φ are particularly relevant as they serve to connect qualitatively different types of model. θ=0 corresponds closely to a well-mixed non-age structured SIR-type model, indicating that without some assortative mixing, for realistic ranges of D and R(t), it is not possible to generate behaviour matching observed influenza time series. Likewise, φ=1 closely approximates a single-strain system, suggesting that without multiple strains to increase the effective number of susceptibles, the required behaviour cannot be produced by simple models. Figure 10 underlines this conclusion with results from a set of simpler sub-models: an age-structured model without multiple strains; a 2-strain model without age structure and a simple SIR model with neither age-structure or multiple strains. Fig A shows that for an age-structured model, mean attack rates are generally too low for realistic values of *R*_0_ unless duration of immunity is very short (<3yrs). Appropriate attack rates also fall within strongly biennial regions of behaviour, leading to a low likelihood. For the 2-strain model (Fig B), reasonable attack rates correspond with much longer durations of immunity and again fall in regions of unrealistic behaviour. The simple SIR model generates appropriate mean attack rates at reasonable values of *R*_0_ and D. However, the behaviour in this region, although not strongly periodic, yields annual attach rate distributions with a low likelihood, as illustrated by the time series in Fig. D. Typically, a majority of low attack rate seasons are interspersed by frequent large outbreaks with attack rates around 25%. 

 As a result, we conclude that to generate the behaviour identified in seasonal time series for ranges of parameter values that match those observed, both age multiple strains and assortative mixing among age groups are necessary. However, it must be remembered that the plots shown here do not represent a complete survey of a multi-dimensional parameter space and that other regions of high likelihood may exist.  

## Acknowledgements

We thank Marc Lipsitch for useful discussions.

## Funding information

The authors acknowledge research funding from the European Union FP7 FluModCont and EMPERIE projects, the Bill and Melinda Gates Foundation and the Medical Research Council.

## Competing interests

The authors have declared that no competing interests exist.

## Supplementary Information

### Data

 To assess the similarity between model output and time series data, we need to information about the distributions for epidemic duration and attack rate. For simplicity, a normal distribution is assumed for both these features. We define duration as the number of weeks in the year for which the incidence rate is higher than a given threshold. For the French ILI data, using a weekly threshold of 100 cases per 100,000 individuals, we recover a mean duration of approximately 11 weeks with a standard deviation of 2 weeks. A similarly defined threshold is used to calculated epidemic durations within the model. As discussed in the Methods Section, the underlying attack rate is difficult to estimate. However, standard deviation of attack rate as a fraction of the mean can be assessed using reported incidence data as a proxy. Data sets of ILI from the UK (1989-2004) and France (1985-2009) were adjusted to remove linear trends. A ‘reported’ attack rate was calculated for each year as the total recorded incidence in a six month period between November and April. Both data sets yielded ratios of standard deviation to mean of approximately 45%. The mean reported annual attack rate for French sentinel data is approximately 5% [11]. Data from household contact studies in France indicate that around 50% of secondary cases consult a GP [25], suggesting that an underlying attack rate of 10% is highly plausible.

### Construction of Pseudo-likelihoods

 We define target probability distributions, f and g, for attack rates and durations respectively based on ILI incidence data, as above. Epidemic durations and attack rates from the model for each year are assigned to discrete bins by number of weeks (52 bins) and percentage of population affected (100 bins), giving counts τ_i_ and A_j_, respectively. Durations and rates were taken from 80 consecutive years of the model run, after a burn-in period to allow the model to stabilize to its long term behaviour. Probabilities for the discrete bins, f_j_ and g_i_, are calculated from the target distributions. We assume that counts τ_i_ and A_j_ are the result of independent draws from multinomial distributions f_j_ and g_i_, giving likelihoods, 

\begin{equation*}\[\begin{align}  & L{{L}_{\tau }}=\sum\limits_{i=0}^{51}[{{\tau_i}\ln ({f_i})-\ln(\tau_i!)}], \\  & L{{L}_{A}}=\sum\limits_{j=0}^{99}[{{{A}_{j}}\ln ({{g}_{j}})-\ln(A_j!)}] \\ \end{align}\]\end{equation*}

Combinatorial terms are included to enable the comparison of different distributions of counts. The total likelihood is the weighted sum of these, 

\begin{equation*}L{{L}_{Tot}}=L{{L}_{\tau }}+wL{{L}_{A}}\end{equation*}

(The weighting factor, *w*, is chosen to give approximately equal ranges of variation to the two pseudo-likelihoods). The above formulation has the advantage that it gives a higher likelihood to parameter sets that generate durations and attack rates that match the shape of the distributions and thus implicitly penalizes highly regular periodic behaviour, such as pure annual and biennial cycles. 

### Model Structure

 The model is, at heart, a deterministic SIR compartmental model with waning immunity. The total simulated population was 60 million, approximating the population of the UK. With 2 strains, however, there are four rather than two immune categories; completely susceptible (S_0_), immune to only one strain (S_1_ or S_2_) and immune to both (S_12_). These compartments are further stratified by age group (subscript a or c for adults or children respectively) and into incubation class for infectives (superscript n for the nth stage). Hence S_1c_ contains adults immune to strain 1 and I_1c_^(2)^ are children infected with strain 1 in incubation stage 2. The dynamics of infected individuals are described by the equations, 

\begin{equation*}\frac{dI_{1\gamma }^{(1)}}{dt}={{\lambda }_{1\gamma }}({{S}_{0}}+{{S}_{2}})-\alpha M.I_{1\gamma }^{(1)}, \end{equation*}

\begin{equation*}\frac{dI_{2\gamma }^{(1)}}{dt}={{\lambda }_{2\gamma }}({{S}_{0}}+{{S}_{1}})-\alpha M.I_{2\gamma }^{(1)}, \end{equation*}

\begin{equation*}\frac{dI_{\delta \gamma }^{(n)}}{dt}=\alpha M(I_{\delta \gamma }^{(n-1)}-I_{\delta \gamma }^{(n)}). \end{equation*}

where γ is c or a and n =2,...,M . *λ_jγ_* is the force of infection of strain *j* on age group γ. These dynamics give rise to gamma-distributed generation time with mean 1/α and shape parameter *M*.  The dynamics of immunity for children are described by the equations 

\begin{equation*}\frac{d{{S}_{0c}}}{dt}=\mu N-{{S}_{0c}}({{\lambda }_{1c}}+{{\lambda }_{2c}})+\sigma ({{S}_{1c}}+{{S}_{2c}})-\frac{{{S}_{0c}}}{{{L}_{c}}}, \end{equation*}

\begin{equation*}\frac{d{{S}_{1c}}}{dt}=(1-\phi ){{S}_{0c}}{{\lambda }_{1c}}-{{S}_{1c}}{{\lambda }_{2c}}-\sigma {{S}_{1c}}+\sigma {{S}_{12c}}-\frac{{{S}_{1c}}}{{{L}_{c}}},\end{equation*}

\begin{equation*}\frac{d{{S}_{2c}}}{dt}=(1-\phi ){{S}_{0c}}{{\lambda }_{2c}}-{{S}_{2c}}{{\lambda }_{1c}}-\sigma {{S}_{2c}}+\sigma {{S}_{12c}}-\frac{{{S}_{2c}}}{{{L}_{c}}},\end{equation*}

\begin{equation*}\frac{d{{S}_{12c}}}{dt}=\phi {{S}_{0c}}({{\lambda }_{1c}}+{{\lambda }_{2c}})+{{S}_{2c}}{{\lambda }_{1c}}+\beta {{S}_{1c}}{{\lambda }_{2c}}-2\sigma {{S}_{12c}}-\frac{{{S}_{12c}}}{{{L}_{c}}}, \end{equation*}

where σ=1/D. For adults, an analogous set apply, but without the birth term and with each compartment ‘fed’ from the corresponding children’s immune class in the standard manner [26]. A schema for the acquisition of immunity is shown in Figure 2. The parameter φ takes the model from two independent strains (φ=0) to effectively a single strain (φ=0), complicated by the two-stage recovery of susceptibility. 

Force of infection is dependent on the nature of mixing between the age groups and on the susceptibilities and infectivities of the different age classes. In practice, resolving the separate effects of susceptibility, infectivity and patterns of contact from data is very difficult. However, large-scale, direct studies of contact patterns indicate that contact between age groups is highly assortative and more intense in school and home than elsewhere [23] [24]. Data indicates a range of approximately 30-50% of cases among children [11] [27]. In our model, we represent the combination of these effects in a mixing matrix, A,  

**Figure:**
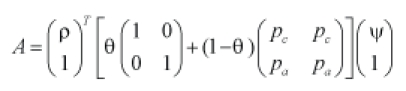

where p_c_ and p_a_ are the fractions of the population in the two age groups and ρ and ψ are the relative susceptibility and infectivity of children, respectively. The central bracketed term allows for variation in the assortativity of mixing between groups as a function of θ, where θ=0 gives uniform random mixing and θ=1 fully assortative mixing. Differences in intensity of contact are parameterized by the first and last terms, representing relative susceptibility and infectivity. The values of ρ and θ given in Table 1 correspond to approximately 40% of cases among children in model runs.   The force of infection experienced by an age group will depend on all aspects of the above mixing matrix as well as the prevalence of infection in the age groups. Forces of infection for children and adults are given by  

\begin{equation*}{{\lambda }_{\delta c}}=\beta \rho \left( \theta \phi \frac{I_{\delta c}^{(M)}}{{{N}_{c}}}+(1-\theta )\frac{(\psi I_{\delta c}^{(M)}+I_{\delta a}^{(M)})}{N} \right)+\varepsilon , \end{equation*}

\begin{equation*}{{\lambda }_{\delta a}}=\beta \left( \theta \frac{I_{\delta a}^{(M)}}{{{N}_{a}}}+(1-\theta )\frac{(\psi I_{\delta c}^{(M)}+I_{\delta a}^{(M)})}{N} \right)+\varepsilon  \end{equation*}

The value of *R*_0_ is controlled via β through the relationship,  

\begin{equation*}{{R}_{0}}=\beta {{e}_{A}}/\alpha \end{equation*}

where e_A_ is the maximum eigenvalue of the matrix A. 

## Tables

**Figure:**
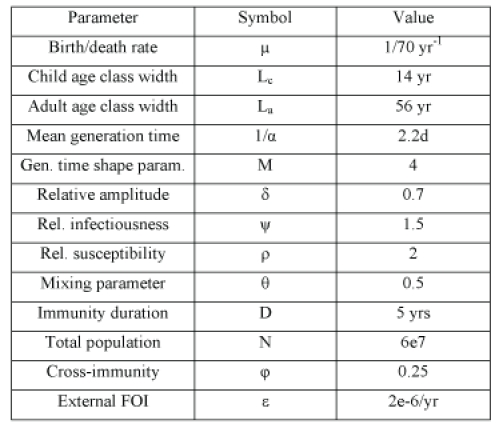
Table 1: Model parameter values. Values given are those used if not otherwise stated 

## Figures

**Figure:**
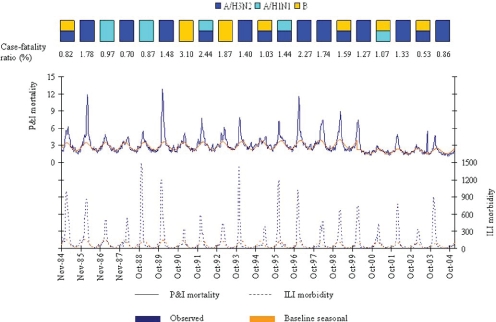
Figure 1: Observed and baseline seasonal rates of P&I mortality and ILI morbidity. The dominant types and subtypes of circulating influenza viruses in France, 1984–2004, are shown in the rectangles [Taken from **[8]**].

**Figure:**
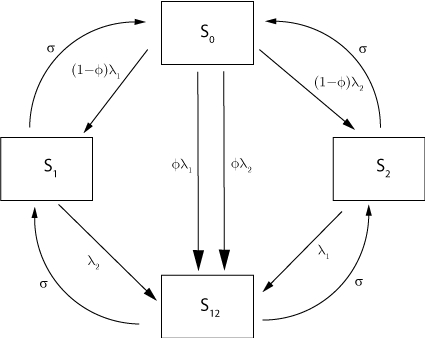
Figure 2: Roots of movement for individuals between immune classes in the 2 strain model.

**Figure:**
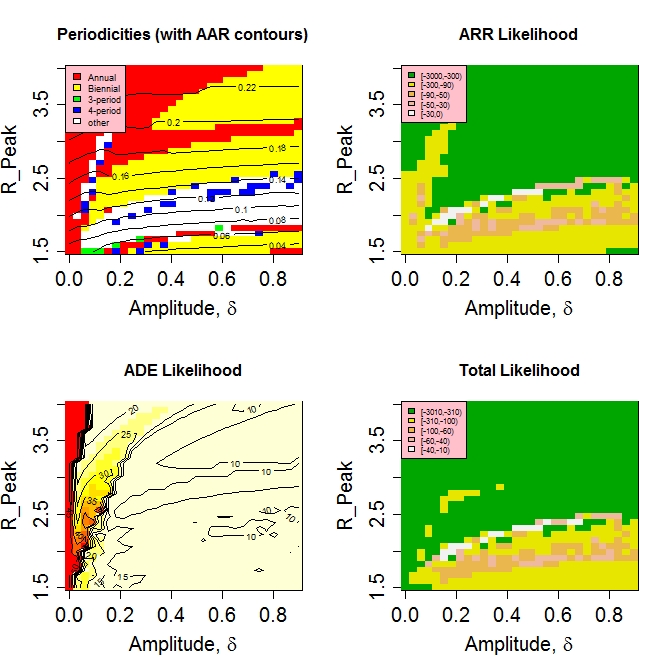
Figure 3: Model behaviour and likelihoods as a function of peak *R_0_* and relative amplitude. Other parameters as 

given in Table 1.

**Figure:**
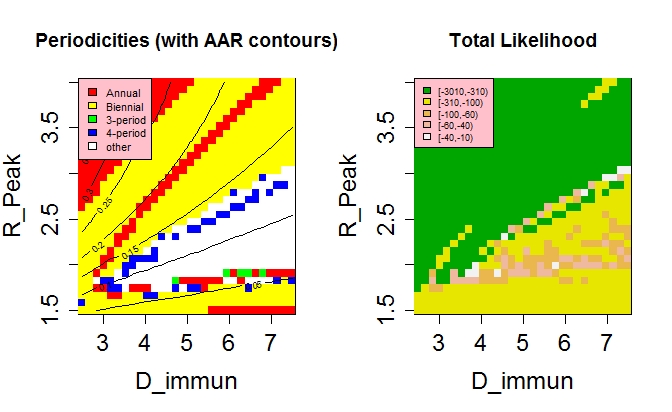


Figure 4: Model behaviour and likelihood as a function of peak *R_0_* and the immune period, D.

**Figure:**
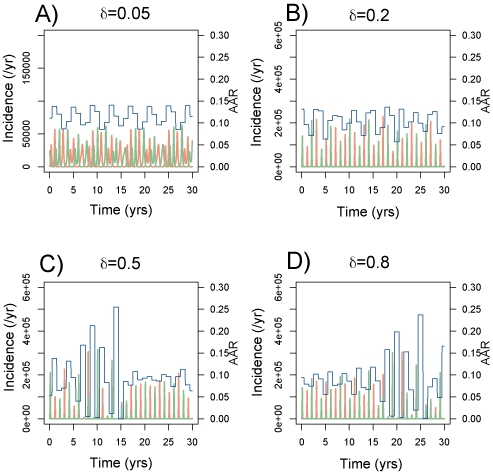

Figure 5: Typical time series for incidence and annual attack rates (piece-wise line) for a range of amplitudes (*R_p_*=2).  Other parameter values as in Table 1.

**Figure:**
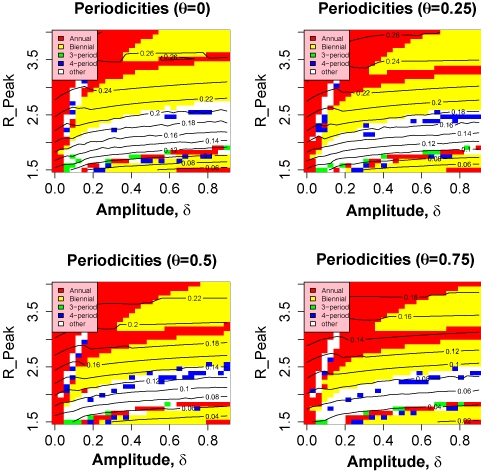
Figure 6: Variation of model behaviour with mixing parameter, θ. Assortativity of mixing increases with θ.

**Figure:**
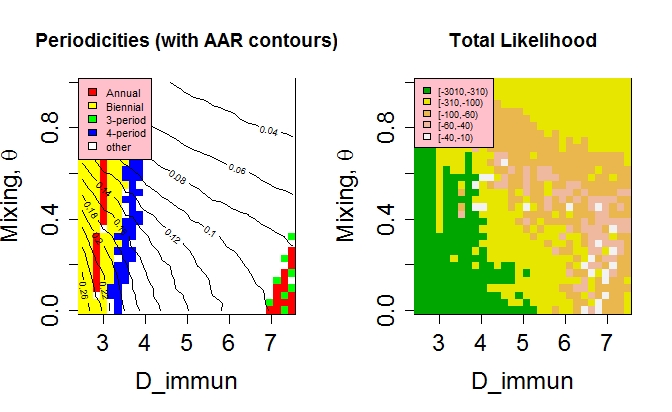

Figure 7: Periodicities and likelihood as a function of the mixing parameter, θ, and length of immunity, D, for peak *R*_0_=2.

**Figure:**
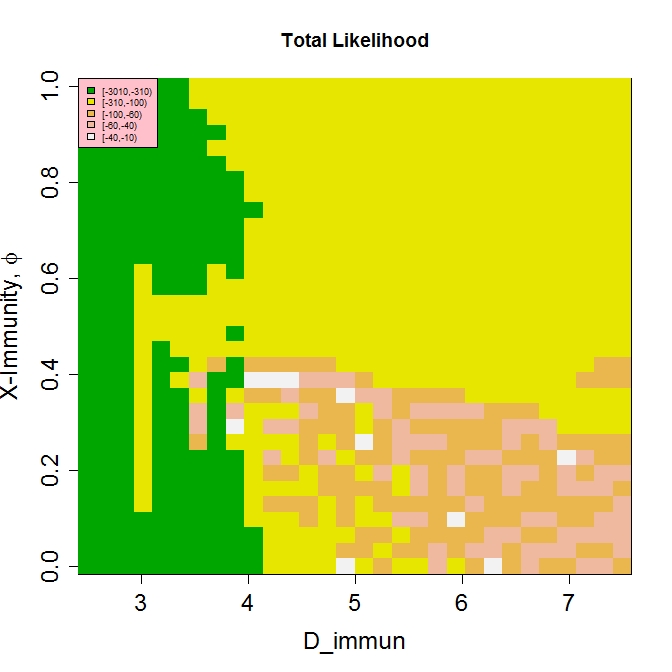
Figure 8: Likelihood as a function of cross-immunity, φ, and length of immunity, D. Peak *R*_0_ set at 2.

**Figure:**
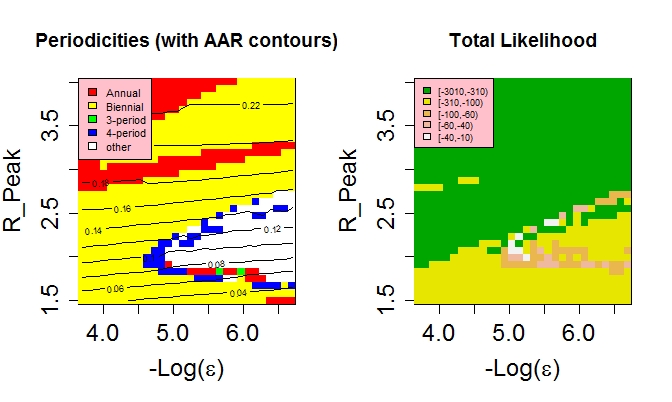

Figure 9: Likelihood as a function of Peak *R*_0_ and the magnitude of the external force of infection.

**Figure:**
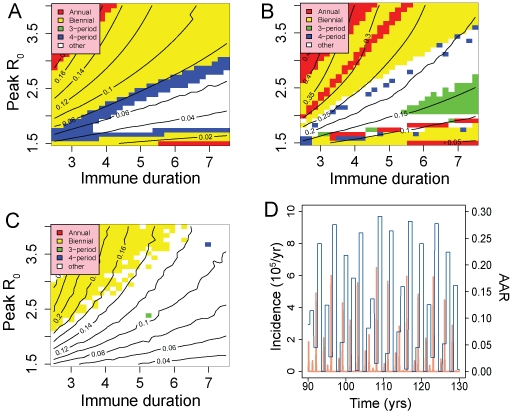
Figure 10: Results from simplified models; A-SIR with age structure, B-SIR with two strains, C and D-simple SIR. Figures A, B and C represent behaviour and mean attack rate as a function of *R*_0_ and Immune duration, D. Fig D shows a time series for the point (R0,D)=(1.9,4) in Fig. C.
